# Supplementary material for: Revisiting Assessment of Computational Methods for Hi-C Data Analysis
Source: Int J Mol Sci. 2023 Sep 7;24(18):13814. doi: 10.3390/ijms241813814 (PMC10531246; doi:10.3390/ijms241813814)
Supplement: Supplementary file 1 [file ijms-24-13814-s001.zip › ijms-2554537 Supplemental Figures.pdf]

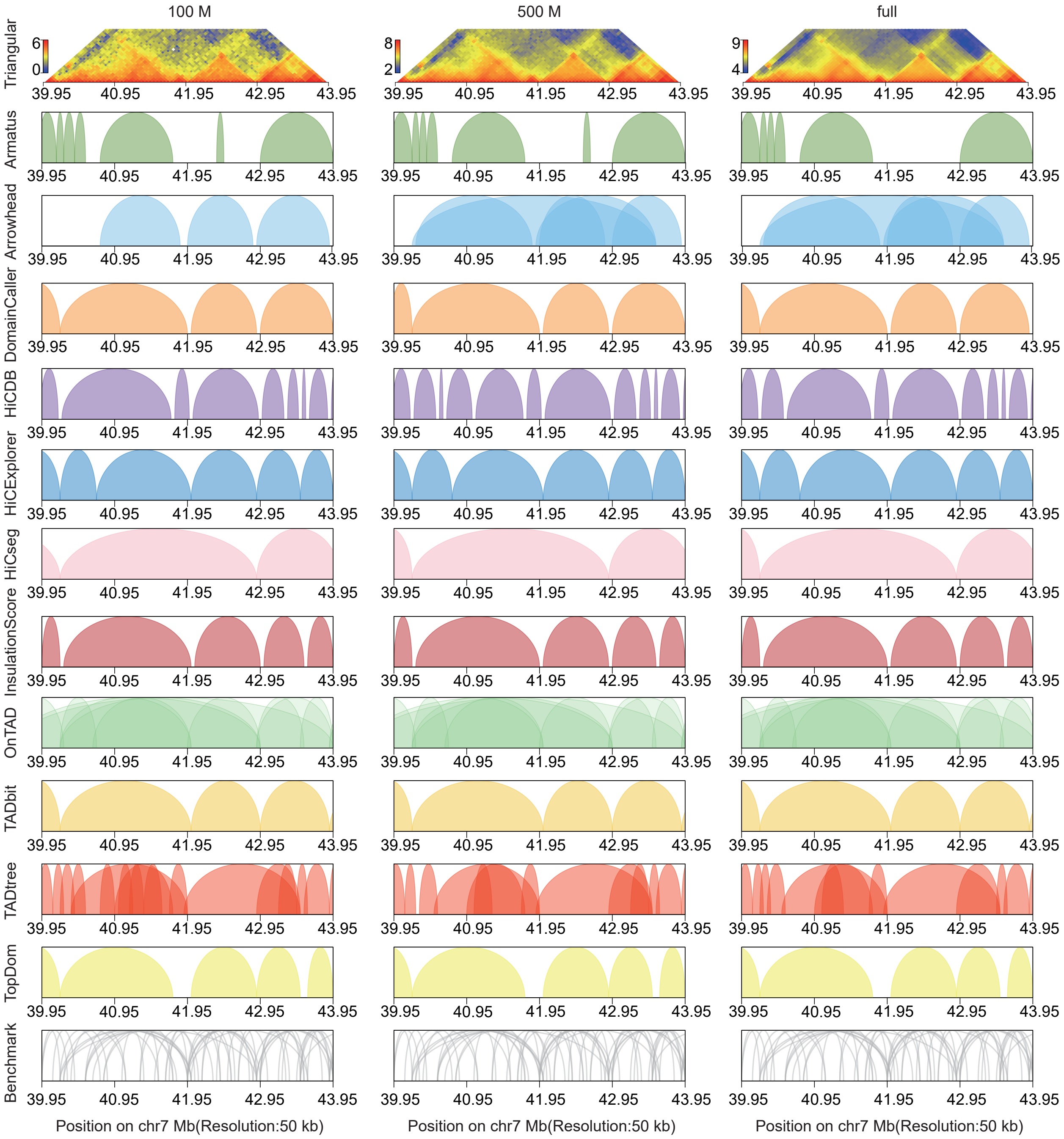

Supplemental Figure S1. TAD detection using 11 tools at resolution of 50 kb and sequencing depths of 100 million read pairs, 500 million read pairs, and the complete GM12878 dataset using the Rao GM12878 dataset (chr7:39,950,000–43,950,000).

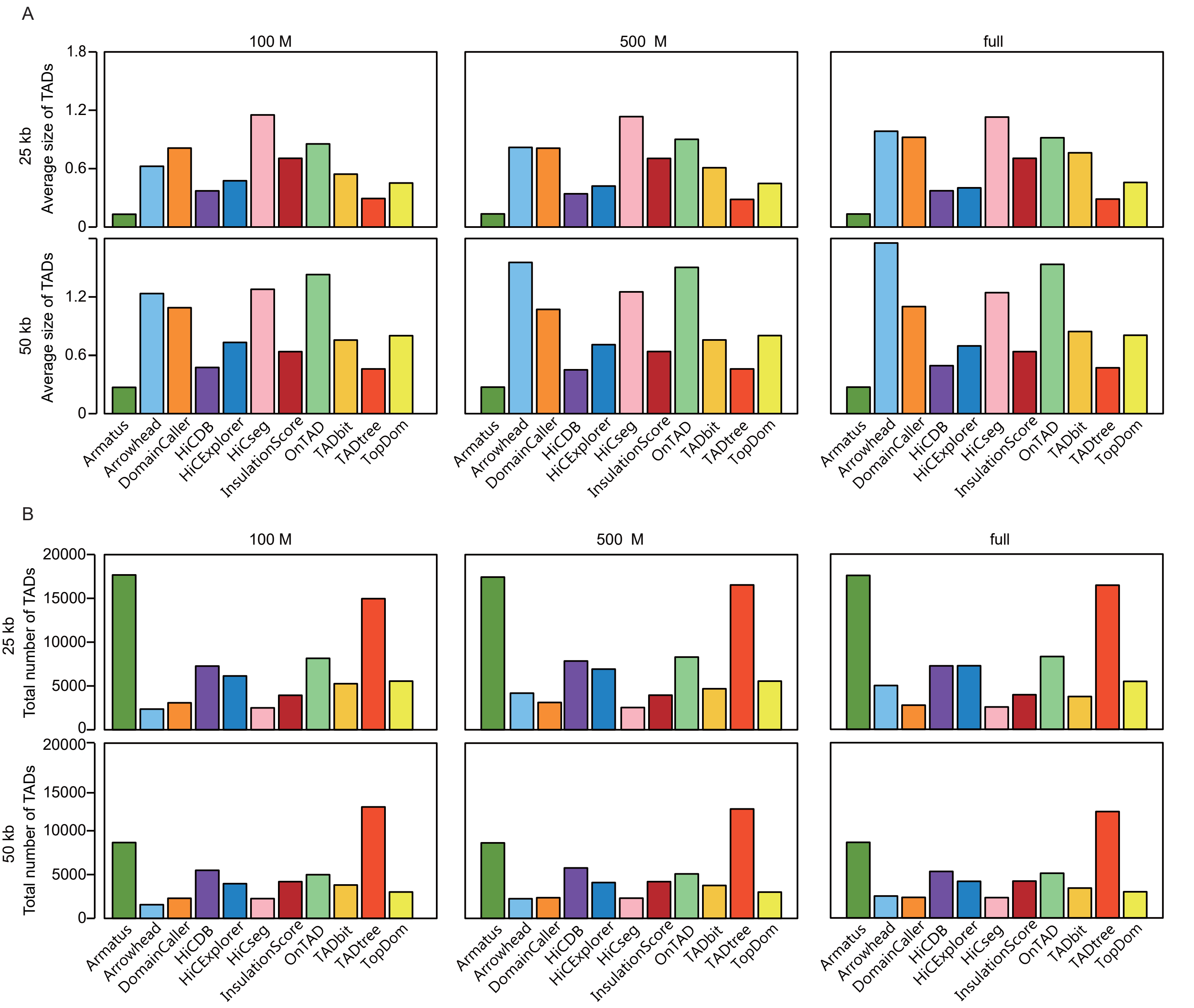

Supplemental Figure S2. TAD prediction statistics of 11 TAD prediction tools at sequencing depths of 100 million read pairs, 500 million read pairs, and the complete GM12878 dataset at 25- and 50-kb resolutions. (**A**) Barplots of the average size of predicted TADs for each program (Supplemental Table S1). (**B**) Barplots of the total number of predicted TADs for each program (Supplemental Table S1).

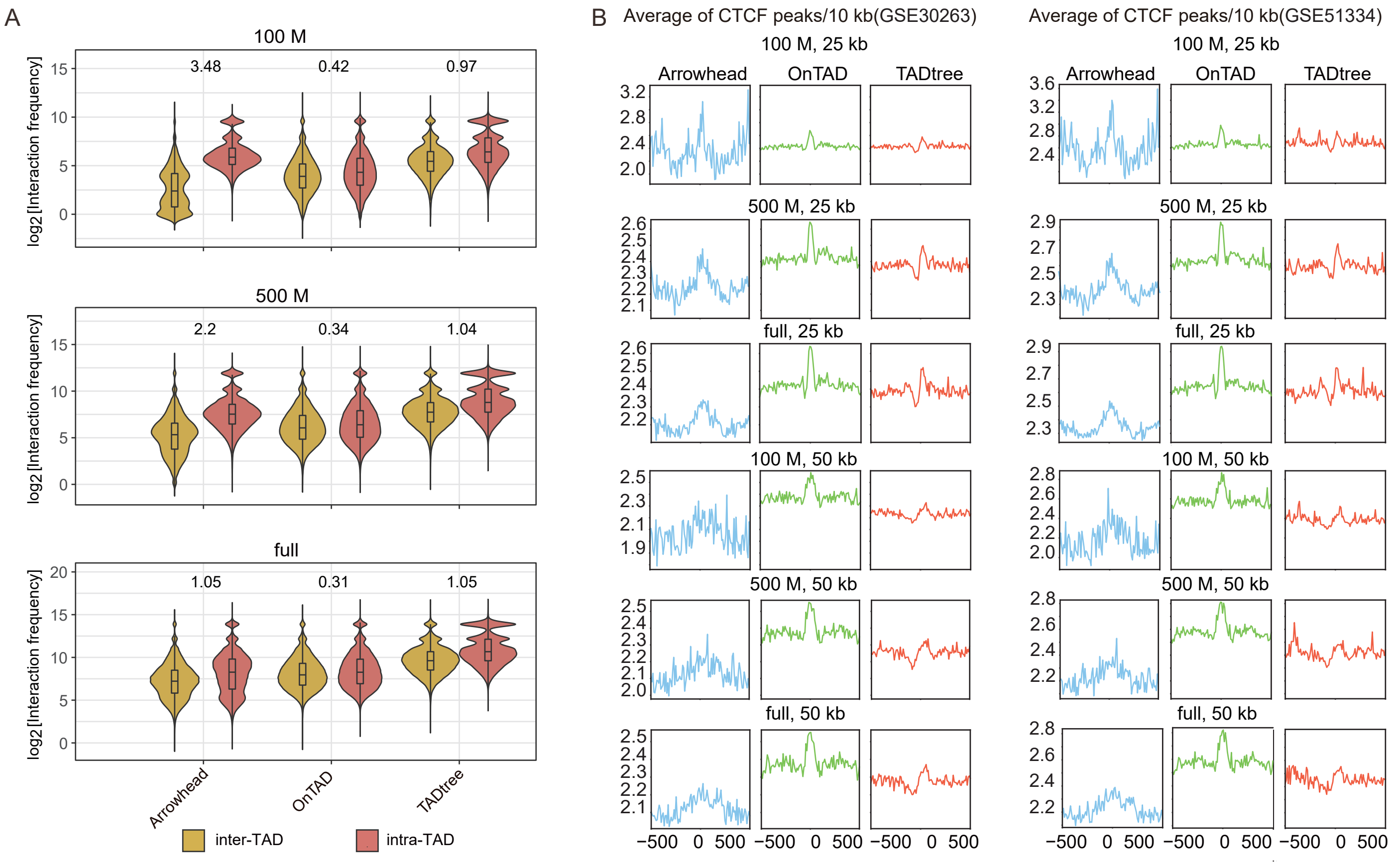

Supplemental Figure S3. Comparison of nested TAD identification tools (Arrowhead, OnTAD and TADtree) evaluated using the Rao GM12878 dataset. **(A)** Violin plots of the within-TAD interaction frequency versus between-TAD interaction frequency for different tools at 50-kb resolution across three sequencing depths. Between-TAD interaction frequency corresponds to pairs of bins that are located in adjacent TADs. Within-TAD interaction frequency corresponds to pairs of bins inside the TADs. The number above each pair of violins represents log_2[fold change], Wilcoxon test was performed for all comparisons, and the *p*-value were less than $2.2e^{-16}$. (**B**) Enrichment of CTCF binding (GSE30263 and GSE51334) in a window of 1 Mb (±500 kb) around the predicted TAD boundaries at three levels of sequencing depths and two levels of resolutions.

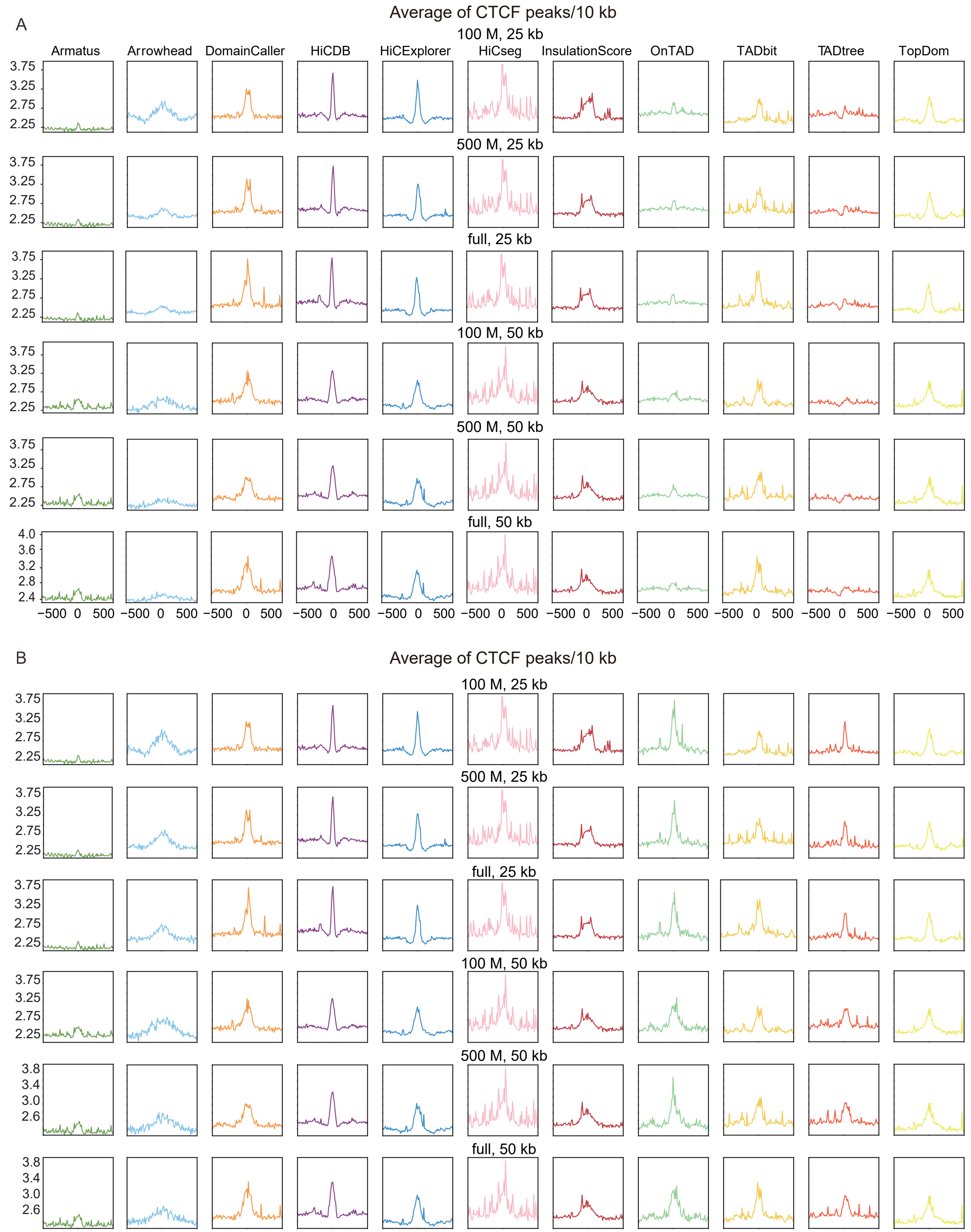

Supplemental Figure S4. Comparison of TAD identification tools evaluated using the Rao GM12878 dataset and CTCF binding dataset (GSE51334). (**A**) Enrichment of CTCF binding in a window of 1 Mb (±500 kb) around the predicted TAD boundaries at three levels of sequencing depths and two levels of resolutions. (**B)** Enrichment of CTCF binding in a window of 1 Mb (±500 kb) around the predicted outer layer TAD boundaries at three levels of sequencing depths and two levels of resolutions.

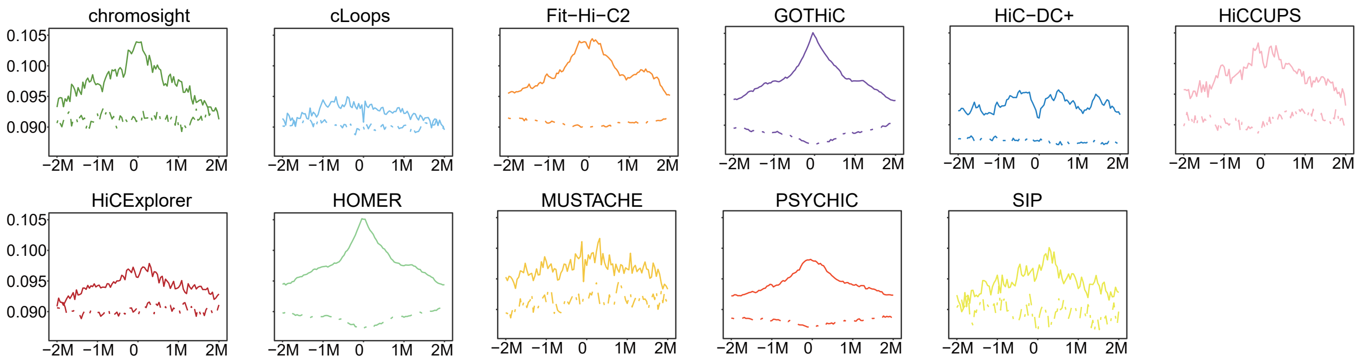

Supplemental Figure S5. Enrichment of conservation in a 4-Mb window (± 2-Mb) around the putative enhancers identified for promoter–enhancer interactions.The solid line represents putative enhancers and the dashed line represents random enhancers.
