# Supplementary material for: Revisiting Assessment of Computational Methods for Hi-C Data Analysis
Source: Int J Mol Sci. 2023 Sep 7;24(18):13814. doi: 10.3390/ijms241813814 (PMC10531246; doi:10.3390/ijms241813814)
Supplement: Supplementary file 1 [file ijms-24-13814-s001.zip › ijms-2554537 Supplemental Methods.pdf]

Supplemental Methods

Supplemental Note 1: Methods for data preprocessing

1.1. distiller-nf

distiller-nf (version 0.3.3) is an end-to-end pipeline, including scripts designed for Hi-C data pre-processing and generation of binned 2D map matrices. In brief, Hi-C forward and reverse reads were aligned to the reference genome separately as single-end reads using bwa (version 0.7.15) [1]; then aligned single-end reads in sam format were parsed and paired, and PCR duplicates were further removed; in the end, valid contacts were aggregated into binned 2D matrices of Hi-C interactions and normalized by iterative correction and eigenvector decomposition (ICE) algorithm from Imakaev et al. [2].

1.2. HiCExplorer

HiCExplorer (version 3.4.1)[3] can be successfully applied with bwa, bowtie2 or hisat2, here we used bowtie2 to align each mate of individually to avoid mapper specific heuristics designed for standard paired-end libraries. The mapped mates were paired and filtered to exclude potentially erroneous reads, such as unmappable reads, self-ligated reads, dangling-ends, PCR duplicates or incomplete digestions. The valid contacts were aggregated into binned 2D matrices based on fixed size bins, and biases were removed from the matrices using the KR matrix balancing algorithm implemented by Juicer (version 1.5.6)[4].

1.3. HiC-Pro

HiC-Pro [5] is a pipeline designed to process Hi-C data from raw reads to normalized contact maps. HiC-Pro aligns reads, filters invalid ligation products, carries out quality controls and produces 2D contact maps. It also implements a fast and memory-efficient module of the ICE method for contact map normalization.

1.4. HOMER

HOMER (version 4.11) [6] trims the FASTQ files, aligns them to the reference genome, and then assemblies them into a HOMER-style tag directory. A "background model" that saves important parameters from normalization is created, which accounts for sources of technical bias in Hi-C interaction counts such as read depth, linear distance, sequencing bias and chromatin compaction. HOMER further creates contact matrices from tag directories, and the "Hi-C background model" is applied to efficiently normalize and calculate expected interaction counts.

1.5. Juicer

Juicer (version 1.5.6)[4] is a platform for analyzing kilobase resolution Hi-C data, which includes a pipeline for generating Hi-C maps from FASTQ raw data files. KR matrix balancing algorithm is implemented by Juicer for matrix normalization.

1.6. TADbit

TADbit (version 1.0)[7] is a computational package that deals with 3C-based data (more specifically Hi-C data), and it includes a pipeline of different steps: preprocessing of paired-end reads from Hi-C experiment, alignment of the reads using GEM, filtering of the mapped reads, and construction of interaction matrices and normalization. ICE algorithm from Imakaev et al. [2] is used for normalization here.

Supplemental Note 2: Methods for the analysis of topologically associating domains

2.1. Data preprocessing

Distiller-nf (version 0.3.3) was applied and the aligned, paired and duplicate-

removed reads were aggregated into binned 2D matrices of Hi-C interactions in 25- and 50-kb resolutions. The intrinsic bias of the matrices, possibly originated from uneven fragment length, GC content and mappability, were removed based on implicit KR matrix balancing algorithm implemented by Juicer[4].

2.2. *HiCseg*

HiCseg (version 1.1)[8], an R package, takes raw or normalized Hi-C contact matrices as input, and performs 2D segmentation using maximum likelihood method to split the genome into TAD regions. Unlike many other TAD detection tools which transforms the 2D matrix information into 1D index, HiCseg directly performs segmentation on the Hi-C matrices using dynamic programming to get the optimal results. HiCseg applies Poisson or negative binomial distribution for raw Hi-C matrices, and Gaussian distribution for normalized data. Given the raw count matrices of our input, the distribution was set to Poisson. As suggested by the author, the maximum number of change-points was set by dividing the chromosome length by 1 Mb to obtain TADs with average length of ~1Mb. Finally, a block-diagonal (D) model was chosen.

2.3. *TADbit*

TADbit (version 1.0)[7] is a Python package with both data pre-processing and TAD calling modules, and we only use the latter in our analysis. We used raw count matrices as input, and normalized the matrices based on a method provided by TADbit, which is similar to the ICE algorithm[7]. TADbit utilizes a breakpoint detection method that partition the genome into TAD regions based on BIC-penalized likelihood. Here, we set the parameter to search centromeric regions to TRUE and leave the other parameters as default.

2.4. *DomainCaller*

DomainCaller (version 1.0)[9], consisting of MATLAB and Perl scripts, is a Hidden Markov Model based partition of the genome using "Directionality Index" (DI). The DI is an index that measure the bias of the upstream and downstream interactions for each Hi-C region, and the maximum distance is normally set to 2 Mb and could be adjusted by the user. We used KR normalized matrices as input and the default parameters of the package.

2.5. *InsulationScore*

The InsulationScore (version 1.0.0)[10] is designed to identify the boundaries between domains by summing up contacts in a sliding window of square shape along the Hi-C matrix diagonal, where regions with low score are considered "insulating", i.e. regions between domains, and those with high scores are postulated as inside domains. We applied this method to KR normalized matrices with default parameters except for insulation square and insulation delta span, which were set to 1 Mb and 200 kb, respectively.

2.6. *Arrowhead*

Arrowhead (version 1.8.9)[4, 11] transforms the patterns of TAD domains from "squares" along the diagonal to "triangles" of high or low signal. Transformation replaces domains with an Arrowhead-shaped motif pointing toward the domain's upper-left corner. The corners of the domains is identified based on specific scores for the "triangles", and used for locating the boundaries of TADs. We applied this method packaged in Juicer (version 1.5.6)[4] to KR normalized matrices with default parameters.

2.7. *TADtree*

TADtree (publicly available at http://compbio.cs.brown.edu/projects/tadtree/ - no version information available (accessed on the 17 June 2021))[12] is an algorithm for the identification of hierarchical topological domains in Hi-C data. This model is based on the

assumption that the increase in contact enrichment with distance is stronger for the inner TAD than for the outer TAD pairs. We used KR normalized matrices as input with default parameters for M, p and q, 6 for N and resolution-adjusted values for S, respectively.

2.8. Armatus

Armatus (version 2.3)[13] uses dynamic programming to obtain alternative optimal and near-optimal domain sets, and identifies a consensus set of domains that persists across various resolutions. To obtain the consensus domains, we used KR normalized matrices as input with gamma-max set to 0.3.

2.9TopDom.

The authors define binSignal(i) as the average contact frequency between an upstream and a downstream chromatin region in a window surrounding bin i, and identify local minima by fitting a piecewise linear curve. The local minima delineate potential domains, whose validity is measured by comparing interactions within and between the domains based on Wilcox rank-sum test. Consistent boundaries supported by deep valleys in the original binSignal(i) curve, and the local minima with significance (p-value < 0.05) for Wilcox rank-sum test are considered as the final set of domain boundaries. We applied this approach (version 0.0.2)[14] to KR normalized matrices with the window size set to 10.

2.10. HiCExplorer

HiCExplorer (version 3.4.1)[3] uses TAD-separation score to measure the degree of separation between the left and right regions at each Hi-C matrix bin. For a running window of different sizes, the TAD-separation score is defined as the mean z-score of all the matrix contacts between the left and right regions (diamond), and TADs are identified as those with a local TAD-separation score minimum. We used KR normalized matrices as input for this software with default parameters except setting ⊚-correctForMultipleTesting fdr'.

2.11. HiCDB/RHiCDB

HiCDB (publicly available at https://github.com/ChenFengling/HiCDB - no version information available (accessed on the 21 June 2021))[15] uses a metric called the local relative insulation (LRI) to assess the insulation strength, which takes into account different window sizes and local background. HiCDB also provides a biologically meaningful LRI cutoff option that considers the CTCF motif enrichment based on a method adapted from gene set enrichment analysis. In brief, HiCDB first orders the candidate domains according to their LRI, and an enrichment score ES is calculated by going through the list. The ES score increases when it encounters a peak with the CTCF motif and decreases otherwise, and the LRI at the maximum ES is chosen as the domain detection cutoff. We applied HiCDB to KR normalized matrices with default parameters.

2.12. OnTAD

OnTAD (version 1.3)[16] calls TADs in two steps. In the first step, the method identifies candidate TAD boundaries using an adaptive local minimum search algorithm with a series of different window sizes ranging from the bin size to the maximum TAD size. In the second step, based on union of the candidate boundaries of all window sizes, OnTAD assembles TADs by identifying pairs of boundaries whose potential TAD area between the boundaries exceeds that of the surrounding area outside of the TAD by a user-defined margin (λ). The dynamic programming algorithm is applied to recursively identify the optimal partition of the genome for obtaining a final hierarchical TAD organization. We applied OnTAD to KR normalized matrices with parameters penalty set to 0.1 and maxsz adjusted for the respective resolutions.

Supplemental Note 3: Methods for the analysis of chromatin interactions

3.1. Data preprocessing

Similar to data preprocessing for TAD identification, distiller-nf (version 0.3.3) was applied and the aligned, paired and duplicate-removed reads were retained for downstream analyses.

3.2. Fit-Hi-C2

Fit-Hi-C2 (version 2.0.7)[17] is designed to identify mid-range intra- and inter-chromosomal contacts in Hi-C data, and we only used its intra-chromosomal function in our analysis. The tool fits a spline to model contact probability values as a function of the average genomic distance for all pairs of interacting locus pairs. The contact probability is then corrected using the bias values by looking up the expected contact probability from the spline. Then a binomial test is carried out using the corrected contact probability and the observed count to obtain a p-value. The resulting p-values are corrected for multiple testing and a Q-value is calculated for each locus pair. As Fit-Hi-C2 has no tunable parameters, we followed the recommended protocol to obtain intra-chromosomal interactions. For comparisons of promoter-enhancer interactions (PEIs) identified by distinct tools in K562, we used 10-kb resolution and Q-value cut-off of 0.05 to obtain the initial set of PEIs [18], which were further filtered by integratively considering Q-value, observed count and observed/expected contact frequency ratio to obtain comparable number of PEIs between Fit-Hi-C2, GOTHiC, HOMER and PSYCHIC. For comparisons of interactions identified by distinct tools in GM12878, we used 5-kb resolution and a Q-value cut-off of 0.05 [18] obtain the initial set of interactions, which were further filtered by integratively considering Q-value, observed count and observed/expected contact frequency ratio to yield comparable number of interactions between Fit-Hi-C2, GOTHiC , HOMER and HiC-DC+.

3.3. GOTHiC

GOTHiC (version 1.22.0)[19] uses a binomial model that corrects the complex combination of known and unknown biases in Hi-C data. The model calculates the probabilities that the observed number of read-pairs are due to random ligations, and produces statistically significant interactions irrespective of genomic distances. For comparisons of promoter-enhancer interactions (PEIs) identified by distinct tools in K562, we used 10-kb resolution and FDR cut-off of 0.05 with observed count over 10 to obtain the initial set of PEIs [18], which were further ranked FDR to obtain comparable number of PEIs between Fit-Hi-C2, GOTHiC, HOMER and PSYCHIC. For comparisons of interactions identified by distinct tools in GM12878, we used 5-kb resolution and a FDR cut-off of 0.05 with observed count over 10 [18] to obtain the initial set of interactions, which were further ranked by FDR and observed count to yield comparable number of interactions between Fit-Hi-C2, GOTHiC, HOMER and HiC-DC+.

3.4. HOMER

HOMER (version 4.11)[6] generates a background model that normalizes interactions for linear genomic distance and coverage at the chosen bin level, which produces the expected read count. The observed count is compared to expected count, and a binomial test is applied to call significant chromatin interactions. For comparisons of promoter-enhancer interactions (PEIs) identified by distinct tools in K562, we used 10-kb resolution and p-value cut-off of 0.001 to obtain the initial set of PEIs [18], which were further filtered by integratively considering FDR and observed/expected contact frequency ratio to obtain comparable number of PEIs between Fit-Hi-C2, GOTHiC, HOMER and PSYCHIC. For comparisons of interactions identified by distinct tools in GM12878, we used 5-kb resolution and a p-value cut-off of 0.001 (ref NM)[18] obtain the initial set of interactions, which were further filtered by integratively considering FDR and observed/expected contact frequency ratio to yield comparable number of interactions between Fit-Hi-C2, GOTHiC, HOMER and HiC-DC+.

3.5. HiCCUPS

HiCCUPS (version 1.22.01)[4, 11] uses KR normalized matrices as default input and identifies enriched pixels with respect to four background neighboring areas based on the peak width and the window size. A modified Benjamini-Hochberg FDR is used for multiple hypothesis testing, and identified significant peaks are further aggregated into clusters when they are in linear genomic proximity. Here, we used default settings that involve combining interactions called at multiple resolutions of 5 kb, 10 kb and 25 kb with default parameters. At all resolutions we set the FDR threshold to 0.1(ref NM)[18] .

3.6. cLoops

cLoops (version 0.93)[20], uses an unbiased clustering algorithm based on a specific improvement to DBSCAN (Density-Based Spatial Clustering of Applications with Noise) to find candidate loops, and a PLB (permutated local background) method is applied for estimation of a candidate loop's statistical significance. We chose recommended option [illegible] m 3' for application of cLoops to Hi-C data, and [illegible]significant' loops designated by cLoops were retained.

3.7. PSYCHIC

PSYCHIC (publicly available at https://github.com/dhkron/PSYCHIC - no version information available (accessed on the 2 March 2022))[21] uses a probabilistic model to segment the genome into hierarchical domains, and fits local background models based on separate domains. Z-test and multiple hypothesis testing were applied to background corrected contact frequencies to estimate the significance of promoter-enhancer interactions (PEIs). All analyses were carried out with default parameters. For comparisons of PEIs identified by distinct tools in K562, we used 10-kb resolution and FDR-value cut-off of 0.05 to obtain the PEIs, which renders comparable number of PEIs between Fit-Hi-C2, GOTHiC, HOMER and PSYCHIC.

3.8. HiC-DC+

HiC-DC+ (version 0.99.13)[22] uses a two-step model fitting strategy to perform negative binomial (NB) regression for estimation of the expected read count in an interaction bin based on genomic distance and the GC content, mappability, and effective bin size in the respective pair of genomic loci. The p-value for each interaction bin is calculated as 1 minus the cumulative distribution function fit, and is adjusted for FDR utilizing the Benjamini–Hochberg procedure to identify significantly interacting bins. All analyses were carried out with default parameters. For comparisons of PEIs identified by distinct tools in K562, we used 10-kb resolution and FDR-value cut-off of 0.05. For comparisons of interactions identified by distinct tools in GM12878, we used 5-kb resolution and a FDR-value cut-off of 0.01 to yield comparable number of interactions between Fit-Hi-C2, GOTHiC, HOMER and HiC-DC+.

3.9. HiCExplorer

HiCExplorer (version 3.4.1)[3] calculates a continuous negative binomial distribution for each genomic linear distance for estimation of significance, and interaction pairs with a p-value less than a specified threshold are accepted. In a second step, each candidate is compared to its neighborhood defined by the a user-selected window size in the 2D dimensions on the contact heat-map. Only the candidate with the highest peak values is retained for each neighborhood. In the last step, the neighborhood is segregated into a peak and background region based on a custom peak width. We used recommended parameters for all resolutions (i.e., a windowSize of 10 and a peakWidth of 6 were chosen for 10-kb matrices), and retained loops using a FDR-value cut-off of 0.05.

3.10. chromosight

chromosight (version 1.3.3)[23] applies a balancing normalization procedure to a whole-genome contact map for attenuation of experimental biases, and adjusted each pixel by its expected value estimated by the polymeric behaviour (the distance-dependent

contact decay). A template (kernel) representing a particular 3D structure (i.e. a loop) is compared to sub-images divided from the complete heat-map, and the sub-images with the highest correlation values are labelled as potential matches. Finally, the maximum within each correlation focus is extracted and considered as conforming to a particular 3D structure (i.e. a loop). Default parameters were used for all resolutions and a FDR-value cut-off of 0.05 was applied to retain the loops.

3.11. SIP

SIP (version 1.6.1)[24] applies a Gaussian blur, contrast enhancement, white top-hat, and a minimum-maximum filter to Hi-C contact maps, and obtains a corrected image of the interactions, which is used with a regional maxima detection algorithm to detect a candidate loops. Candidate loops must then pass 6 filters that utilize the local background based on the original distance-normalized contact map, and finally, an empirical FDR of significance is computed as the enrichment of loops over random sites at equal distances. Default parameters were used for all resolutions and a FDR-value cut-off of 0.05 was applied to retain the loops.

3.12. MUSTACHE

MUSTACHE (version 1.0.2)[25] repeatedly convolves the initial contact map with increasing 2D Gaussians and obtains a scale-space representation of the image. Pairwise neighboring Gaussian images are subtracted, creating the difference-of-Gaussian (DoG) images. By contrasting each pixel to its $3 \times 3 \times 3$ neighborhood in DoG image space, the local maxima are identified as the final loop set. Default parameters were used for all resolutions and a FDR-value cut-off of 0.05 was applied to retain the loops.

Reference

1. Li H, Durbin R. Fast and accurate short read alignment with Burrows-Wheeler transform, Bioinformatics 2009;25:1754-1760.
2. Imakaev M, Fudenberg G, McCord RP et al. Iterative correction of Hi-C data reveals hallmarks of chromosome organization, Nat Methods 2012;9:999-1003.
3. Ramírez F, Bhardwaj V, Arrigoni L et al. High-resolution TADs reveal DNA sequences underlying genome organization in flies, Nat Commun 2018;9:189.
4. Durand NC, Shamim MS, Machol I et al. Juicer Provides a One-Click System for Analyzing Loop-Resolution Hi-C Experiments, Cell Syst 2016;3:95-98.
5. Servant N, Varoquaux N, Lajoie BR et al. HiC-Pro: An optimized and flexible pipeline for Hi-C data processing, Genome Biol 2015;16:259.
6. Heinz S, Benner C, Spann N et al. Simple combinations of lineage-determining transcription factors prime cis-regulatory elements required for macrophage and B cell identities, Mol Cell 2010;38:576-589.
7. Serra F, Baù D, Goodstadt M et al. Automatic analysis and 3D-modelling of Hi-C data using TADbit reveals structural features of the fly chromatin colors, PLoS Comput Biol 2017;13:e1005665.
8. Lévy-Leduc C, Delattre M, Mary-Huard T et al. Two-dimensional segmentation for analyzing Hi-C data, Bioinformatics 2014;30:i386-392.
9. Dixon JR, Selvaraj S, Yue F et al. Topological domains in mammalian genomes identified by analysis of chromatin interactions, Nature 2012;485:376-380.
10. Crane E, Bian Q, McCord RP et al. Condensin-driven remodelling of X chromosome topology during dosage compensation, Nature 2015;523:240-244.
11. Rao SS, Huntley MH, Durand NC et al. A 3D map of the human genome at kilobase resolution reveals principles of chromatin looping, Cell 2014;159:1665-1680.
12. Weinreb C, Raphael BJ. Identification of hierarchical chromatin domains, Bioinformatics 2016;32:1601-1609.
13. Filippova D, Patro R, Duggal G et al. Identification of alternative topological domains in chromatin, Algorithms Mol Biol 2014;9:14.
14. Shin H, Shi Y, Dai C et al. TopDom: An efficient and deterministic method for identifying topological domains in genomes, Nucleic Acids Res 2016;44:e70.
15. Chen F, Li G, Zhang MQ et al. HiCDB: A sensitive and robust method for detecting contact domain boundaries, Nucleic Acids Res 2018;46:11239-11250.
16. An L, Yang T, Yang J et al. OnTAD: Hierarchical domain structure reveals the divergence of activity among TADs and boundaries, Genome Biol 2019;20:282.
17. Kaul A, Bhattacharyya S, Ay F. Identifying statistically significant chromatin contacts from Hi-C data with FitHiC2, Nat Protoc 2020;15:991-1012.
18. Forcato M, Nicoletti C, Pal K et al. Comparison of computational methods for Hi-C data analysis, Nat Methods 2017;14:679-685.

19. Mifsud B, Tavares-Cadete F, Young AN et al. Mapping long-range promoter contacts in human cells with high-resolution capture Hi-C, Nat Genet 2015;47:598-606.
20. Cao Y, Chen Z, Chen X et al. Accurate loop calling for 3D genomic data with cLoops, Bioinformatics 2020;36:666-675.
21. Ron G, Globerson Y, Moran D et al. Promoter-enhancer interactions identified from Hi-C data using probabilistic models and hierarchical topological domains, Nat Commun 2017;8:2237.
22. Sahin M, Wong W, Zhan Y et al. HiC-DC+ enables systematic 3D interaction calls and differential analysis for Hi-C and HiChIP, Nat Commun 2021;12:3366.
23. Matthey-Doret C, Baudry L, Breuer A et al. Computer vision for pattern detection in chromosome contact maps, Nat Commun 2020;11:5795.
24. Rowley MJ, Poulet A, Nichols MH et al. Analysis of Hi-C data using SIP effectively identifies loops in organisms from C. elegans to mammals, Genome Res 2020;30:447-458.
25. Roayaei Ardakany A, Gezer HT, Lonardi S et al. Mustache: Multi-scale detection of chromatin loops from Hi-C and Micro-C maps using scale-space representation, Genome Biol 2020;21:256.

.
