# Supplementary material for: Revisiting Assessment of Computational Methods for Hi-C Data Analysis
Source: Int J Mol Sci. 2023 Sep 7;24(18):13814. doi: 10.3390/ijms241813814 (PMC10531246; doi:10.3390/ijms241813814)
Supplement: Supplementary file 1 [file ijms-24-13814-s001.zip › ijms-2554537 supplemental Tables.pdf]

Supplemental Table S1. Details of input, functionality, hierarchical TAD structure, language, and availability for each tool used in this comparison.

	Method	Input	Functionality	Hierarchical TAD structure	Language	Availability	access date
TADs	Armatus	n x n interaction matrix -gzipped	Calls TADs on raw and normalized matrices	No	C++	https://github.com/kingsfordgroup/armatus	8 June 2021
	Arrowhead	hic format(Not from interaction matrix)	Calls TADs-parts of JuiceBox which has several other functionalities	Yes	Java	https://github.com/aidenlab/juicer/wiki/Arrow-head	15 June 2021
	DomainCaller	n x (n+3) interaction matrix	Calls TADs	No	Matlab、Perl	https://github.com/XiaoTaoWang/domaincaller	2 February 2021
	HiCDB	dense(n x n interaction matrix) or sparse (a K x 3 table)	Calls TADs	No	Matlab	https://github.com/ChenFengling/HiCDB	21 June 2021
	HiCExplorer	h5 format	Calls TADs, creation contact matrice, correction contacts, A/B compartments, merging, reordering or chromosomes, conversion from different formats	No	Python	https://hicexplorer.readthedocs.io/en/latest/	1 August 2020
	HiCSeg	n x n interaction matrix	Calls TADs	No	R、C	https://cran.r-project.org/web/pack-ages/HiCseg/index.html	19 June 2021
	InsulationScore	n x n interaction matrix	Call TADs	No	Perl	https://github.com/dekkerlab/crane-nature-2015	1 February 2021
	OnTAD	n x n interaction matrix	Call TADs	Yes	C++	https://github.com/anlin00007/OnTAD	8 February 2020
	TADbit	n x n interaction matrix	Maps reads, normalizes and plots IF matrices.Calls and plots TADs. TAD clustering. 3D modelling.	No	Python	https://github.com/3DGenomes/TADbit	15 June 2021
	TADtree	n x n interaction matrix	Calls TADs	Yes	Python	Raphael Lab // TADTree (brown.edu)	17 June 2021
	TopDom	n x (n+3) interaction matrix	Calls TADs	No	R	http://zhoulab.usc.edu/TopDom/	15 July 2019
Chromatin interactions	chromosight	cool format	Detect chromatin loops	—	Python	https://chromosight.readthedocs.io	24 January 2021
	cLoops	BEDPE format	Detect chromatin loops	—	Python	https://github.com/YaqiangCao/cLoops	5 February 2021
	Fit-Hi-C2	hic format	Find chromtain significant interactions	—	python、R	https://github.com/ay-lab/fithic	11 November 2020
	GOTHiC	mapped read pairs in bam for-mat	Find chromtain significant interactions	—	R	http://bioconductor.org/packages/re-lease/bioc/html/GOTHiC.html	26 May 2021
	HiC-DC+	hic format	Find chromtain significant interactions	—	R	https://github.com/mervesa/HiCDCPlus/	19 May 2021
	HiCCUPS	hic format	Detect chromatin loops	—	Java	https://github.com/aidenlab/juicer	11 January 2021
	HiCExplorer	cool format	Calls TADs, creation contact matrice, correction contacts, A/B compartments, merging, reordering or chromosomes, conversion from different formats	—	Python	https://hicexplorer.readthedocs.io/en/latest/	1 August 2020

HOMER	mapped read pairs in sam format	Motif Discovery and ChIP-Seq analysis	—	C++ 、perl	http://homer.ucsd.edu/homer/download.html	19 August 2020
MUSTACHE	a contact map and a normalization/bias vector, hic format or cool format	Detect chromatin loops	—	Python	https://github.com/ay-lab/mustache/	10 June 2021
PSYCHIC	cool format	Finding putative enhancers	—	Python	https://github.com/dhkron/PSYCHIC	2 March 2021
SIP	hic format	Detect chromatin loops	—	Java	https://github.com/PouletAxel/SIP	25 January 2021

Supplemental Table S2. Number of TADs.

Evaluation type	Dataset	Method	200M		1000M		full	
			25kb	50kb	25kb	50kb	25kb	50kb
TADs	GM12878	Armatus	17677	8654	17427	8620	17572	8626
		Arrowhead	2358	1561	4178	2247	4996	2493
		DomainCaller	3076	2295	3121	2361	2758	2347
		HiCDB	7267	5486	7844	5754	7246	5299
		HiCExplorer	6147	3964	6933	4093	7259	4168
		HiCSeg	2501	2254	2538	2302	2550	2317
		InsulationScore	3941	4191	3945	4186	3941	4195
		OnTAD	8161	4992	8298	5076	8305	5091
		TADbit	5263	3805	4684	3758	3747	3411
		TADtree	14974	12720	16534	12495	16456	12132
		TopDom	5549	3007	5554	2999	5472	2986

Supplemental Table S3. Details of CTCF , H3K27ac ChIP-seq and DNase-seq data used in this study.

Experiment	Cell types	Accession number
CTCF peaks	GM12878	GSE51334<br>GSE30263<br>GSE29611
CTCF peaks	K562	GSE70482
DNase peaks	K562	GSE25344
H3K27ac peaks	K562	GSE107726

Supplemental Table S4. Number of interactions and PEIs in two datasets.

Evaluation type	Dataset	Method	PEIs	Interactions
Chromatin interactions	GM12878	chromosight	2387	17132
		cLoops	2244	25057
		Fit-Hi-C2	52800	300000
		GOTHiC	60945	339438
		HiC-DC+	26285	276963
		HiCCUPS	1740	11229
		HiCExplorer	7664	47272
		HOMER	52014	300000
		MUSTACHE	1715	12608
		SIP	1339	9675
Promoter-enhancer interactions	K562	chromosight	1923	9436
		cLoops	3978	11442
		Fit-Hi-C2	124052	475710
		GOTHiC	131863	683367
		HiC-DC+	22868	148828
		HiCCUPS	1419	5098
		HiCExplorer	3422	17296
		HOMER	126850	618485
		MUSTACHE	837	4059
		PSYCHIC	131051	131051
		SIP	705	3713

Supplemental Table S5. Rankings based on distinct evaluation metrics for comparison of preprocessing methods.

Method	Alignment and filter	Reproducibility	
		Raw	Normalized
distiller-nf	**1**	5	2
Juicer	2	4	3
HiC-Pro	5	**1**	**1**
HiCExplorer	6	6	6
HOMER	4	3	5
TADbit	3	2	4

* Smaller number indicating better performance.

Supplemental Table S6. Rankings based on distinct evaluation metrics for comparison of TAD identification methods.

Method	Positive predictive value		Reproducibility				$\log_2$[interaction frequencies]			Enrichment of CTCF binding
			TAD interval		TAD boundary					
	TAD interval	TAD boundary	Across two resolutions	Across three sequencing depths	Across two resolutions	Across three sequencing depths	100M	500M	Full	
Armatus	5	3	10	7	4	6	7	5	6	8
Arrowhead	8	9	9	10	11	11	**1**	8	10	10
DomainCaller	**1**	4	2	5	3	8	2	**1**	**1**	3
HiCDB	11	**1**	11	8	10	3	8	7	8	**1**
HiCExplorer	3	7	5	3	8	5	5	4	5	2
HiCSeg	6	10	**1**	**1**	**1**	2	6	6	7	5
InsulationScore	9	2	3	4	2	**1**	3	2	2	7
OnTAD	7	11	4	6	5	7	11	10	11	9
TADbit	2	8	7	11	9	10	9	11	4	6
TADtree	10	5	8	9	6	9	10	9	9	11
TopDom	4	6	6	2	7	4	4	3	3	4

* Smaller number indicating better performance.

Supplemental Table S7. Rankings based on distinct evaluation metrics for comparison of CI identification methods.

	PEI percentage	True positive interactions
chro-mosight	6	9
cLoops	10	10
Fit-Hi-C2	2	**1**
GOTHiC	**1**	3
HiC-DC+	9	4
HiCCUPS	5	5
HiCEx-plorer	4	7
HOMER	3	2
MUS-TACHE	8	6
SIP	7	8

* Smaller number indicating better performance.

Supplemental Table S8. Rankings based on distinct evaluation metrics for comparison of PEI identification methods.

	PEI percentage	PEI's location at TAD	Precision–recall	conservation	H3K27ac ChIP-Seq	CTCF ChIP-Seq	DNaseSeq
chromosight	7	**1**	5	4	11	10	11
cLoops	<u>2</u>	11	7	11	4	<u>2</u>	5
Fit-Hi-C2	4	5	**1**	3	3	5	**1**
GOTHiC	9	9	3	**1**	<u>2</u>	9	3
HiC-DC+	11	10	4	10	7	7	10
HiCCUPS	3	<u>2</u>	7	6	9	**1**	4
HiCExplorer	8	7	7	8	10	6	8
HOMER	6	8	<u>2</u>	<u>2</u>	**1**	8	<u>2</u>
MUSTACHE	5	3	7	9	5	4	7
PSYCHIC	**1**	4	6	5	6	11	9
SIP	10	6	7	7	8	3	6

* Smaller number indicating better performance.
